# Metabolic Covariance Connectivity of Posterior Cingulate Cortex Associated with Depression Symptomatology Level in Healthy Young Adults

**DOI:** 10.3390/metabo13080920

**Published:** 2023-08-06

**Authors:** Zhixin Wang, Chris Baeken, Guo-Rong Wu

**Affiliations:** 1 Key Laboratory of Cognition and Personality, Faculty of Psychology, Southwest University, Chongqing 400715, China; wzx9898998@email.swu.edu.cn; 2 Faculty of Medicine and Health Sciences, Department of Head and Skin, Ghent Experimental Psychiatry (GHEP) Lab, Ghent University, 9000 Ghent, Belgium; chris.baeken@ugent.be

**Keywords:** posterior cingulate cortex, metabolic covariance connectivity, resting-state networks

## Abstract

Early detection in the development of a Major Depressive Disorder (MDD) could guide earlier clinical interventions. Although MDD can begin at a younger age, most people have their first episode in young adulthood. The underlying pathophysiological mechanisms relating to such an increased risk are not clear. The posterior cingulate cortex (PCC), exhibiting high levels of brain connectivity and metabolic activity, plays a pivotal role in the pathological mechanism underlying MDD. In the current study, we used the (F-18) fluorodeoxyglucose (FDG) positron emission tomography (PET) to measure metabolic covariance connectivity of the PCC and investigated its association with depression symptomatology evaluated by the Centre for Epidemiological Studies Depression Inventory—Revised (CESD-R) among 27 healthy individuals aged between 18 and 23 years. A significant negative correlation has been observed between CESD-R scale scores and the PCC metabolic connectivity with the anterior cingulate, medial prefrontal cortex, inferior and middle frontal gyrus, as well as the insula. Overall, our findings suggest that the neural correlates of depressive symptomatology in healthy young adults without a formal diagnosis involve the metabolic connectivity of the PCC. Our findings may have potential implications for early identification and intervention in people at risk of developing depression.

## 1. Introduction

Major Depressive Disorder (MDD) is a complex psychological disorder in which symptoms in clinic typically manifest as enduring negative emotional states, anhedonia, feelings of hopelessness and behavioral changes, such as decreased activity levels and loss of appetite. In contrast to daily mood fluctuations, the MDD has a high incidence and recurrence rate. During an MDD episode, patients persist in negative cognitive evaluations and emotional experiences, which may result in suicidal behavior [1]. Therefore, the pathogenesis of MDD and early identification of at-risk individuals have become crucial research objectives. Global prevalence of MDD, especially among young adults, has been on the rise in modern times [2]. Although MDD can manifest at an earlier age, people often experience their first episode during young adulthood [3]. According to Statistics and Quality (2015), around 10% of individuals aged 18–25 in the United States experienced a major depressive episode within the previous year [4]. Additionally, approximately 30% of young adults in the United States have encountered such intense feelings of sadness at least once in the past year that it negatively affected their functional abilities [5]. Since young adulthood is a critical stage of role transition and adaptation, characterized by increased independence, stressors, responsibilities and impactful life events [6,7,8], this group has an increased risk of depression. However, the underlying pathogenesis remains unclear.

Brain glucose metabolism may serve as a valuable physiological indicator for assessing neuronal activity and holds great significance in elucidating neurophysiological mechanisms underlying the increased risk of depression in young adults. Pathologic alterations of metabolism in brain structures implicated in emotional/cognitive function in depression have been identified as significant contributors to the pathological mechanisms of MDD [6,7,9,10]. In particular, the posterior cingulate cortex (PCC) exhibits high connectivity and metabolic activation level [8,11,12] in which metabolic activity was indicated to be a significant neuroimaging biomarker of depression symptoms and antidepressant treatment outcome [10]. Specifically, the metabolic changes in the left posterior cingulate gyrus before and after treatment were negatively correlated with the improvement of tension/anxiety symptom factors of depression [13]. Moreover, decreased metabolism in the PCC was closely associated with treatment outcomes for antidepressant medication and cognitive behavior therapy (CBT)-based interventions [14,15]. The PCC plays a crucial role in the investigation of neurophysiological abnormalities associated with an increased risk of depression in young adults. Also in the healthy state, the PCC is involved in regulating emotional and memory-related processes [16], as well as playing a crucial role in internally directed cognition [11,12], which underline its significance in impaired emotional and cognitive processing, the core symptoms of major depression. Therefore, the PCC has garnered considerable attention from researchers, not only due to its involvement in various brain functions, but also because of its pathological implications during MDD episodes.

In addition to the significance of PCC metabolic activity in pathological mechanisms of MDD, recent functional magnetic resonance imaging (fMRI) studies have also highlighted the PCC functional connectivity as potential neural correlates of depression risk. In the healthy as well as in the depressed state, the PCC exhibits extensive functional connectivity with other nodes in the large-scale brain networks, particularly the default mode network (DMN), central executive network (CEN) as well as salience network (SN). These connections are known to be disrupted in MDD, and they significantly correlate with depression severity as evaluated by the Hamilton Rating Scale for Depression (HAMD) [17,18]. In particular, the altered connectivity patterns of DMN, CEN and SN are associated with current and future depression risk in the young adult population [19]. Additionally, the functional connectivity patterns of PCC are disrupted in young adults with MDD who are experiencing their first episode and have not yet received any treatment. Furthermore, altered PCC connectivity with frontal regions and insula in young MDD patients aged 18–23 years can serve as indicators of depressive rumination and impaired cognitive control—core symptoms of MDD [20,21]. Not only that, but the connectivity of the PCC is also indicative of depression risk, even in subclinical depression or healthy populations. In addition, a significant correlation has been reported between the PCC-habenula connectivity and depressive symptomatology in healthy individuals [22]. Importantly, the increased connectivity between the PCC and the anterior subgenual anterior cingulate cortex (sgACC) observed in subclinical depressed individuals were indicative for the severity of depression symptoms as assessed by the Beck Depression Inventory II (BDI-II) [23]—moreover, elevated EEG functional connectivity between the posterior cingulate cortex, part of the DMN, and the sgACC as a neural marker for rumination [24]. Abnormal DMN activity and rumination may select individuals at risk for depression [25]. Taking these findings into consideration, examining the connectivity of PCC is crucial for comprehending the pathogenesis of MDD in young adults, facilitating early identification of individuals at risk for depression in clinic and enabling effective intervention and treatment.

Compared with fMRI, which indirectly measures neuronal activity through the blood oxygenation level–dependent (BOLD) signal, a semi-quantitative non-absolute index [26,27,28,29], the (F-18) fluorodeoxyglucose (FDG) positron emission tomography (PET) brain imaging technology quantitatively measures neuronal activity by capturing glucose uptake in the brain. This enables it to assess neuronal activity more directly and accurately than BOLD-fMRI [30]. The ^18^FDG-PET brain imaging technology can detect regional brain glucose metabolic activation, which has potential significance for understanding the neurophysiological mechanisms underlying the increased risk of MDD in young adults. As such, examining the brain connectivity derived from ^18^FDG-PET to assess metabolic covariance, an index of cerebral metabolism reflects the covariation of cerebral glucose uptake in the entire brain [31,32], providing a comprehensive and robust representation of network-level brain features for depression risk and early identification of MDD. Similar to the intrinsic network structures of the human brain revealed by fMRI, ^18^FDG-PET has also demonstrated the existence of metabolic covariance networks [33]. Over the past few years, spatial covariance analysis of metabolic images has been widely utilized in neurodegenerative diseases, demonstrating its efficacy in objectively evaluating brain connectivity at a network level [34,35]. Optimized metabolic covariance network analysis in recent years has been utilized to elucidate the physiological mechanisms underlying MDD [36,37,38]. For instance, our recent study has demonstrated a significant correlation between metabolic covariance connectivity in the sgACC and depression severity, as measured by the BDI-II [39]. It remains unclear whether the metabolic covariance network of PCC also serves as a potential metabolic neural substrate for the onset of MDD in young adults.

Our study aims to explore the PCC metabolic covariance network based on the Centre for Epidemiological Studies Depression Inventory—Revised (CESD-R) scale in healthy individuals during their young adulthood, providing valuable insights into potential pathological mechanisms underlying the onset risk of MDD in young adults. To be specific, we used PET brain images to construct the PCC metabolic covariance network and analyzed its correlation with CESD-R scores in healthy young subjects. Although we only included non-depressed individuals, consistent with previous studies demonstrating the involvement of PCC metabolic activity and functional connectivity in mood changes and depressive rumination in MDD as well as in non-depressed samples [13,18,23], we hypothesized that the metabolic covariance connectivity of PCC would primarily include areas within the core brain networks (DMN, CEN and SN) and would correlate significantly with CESD-R scores, suggesting a potential risk of depression symptomatology.

## 2. Materials and Methods

### 2.1. Participants

This study employed data from the Monash rsPET-MR dataset (https://openneuro.org/datasets/ds002898/, accessed on 1 February 2021), which has been previously published in Scientific Data [40]. The dataset consisted of 27 healthy subjects aged between 18 and 23 years, all right-handed, with a mean age of 19 years and education ranging from 13 to 18 years (mean: 14 years). Demographic data are presented in Table 1. All study procedures were approved by the Human Research Ethics Committee of the Monash University. All the participants had normal or corrected-to-normal vision, no history of diagnosed Axis-1 mental illness, diabetes or cardiovascular illness. They also underwent an evaluation for claustrophobia, non-MRI compatible implants, as well as clinical or research PET scans within the preceding 12 months before the scan. Pregnancy screening was conducted for female participants.

Participants were instructed to follow a high protein/low sugar diet for 24 h, fast for 6 h and drink 2–6 glasses of water prior to the PET scan. They also completed a brief cognitive battery and demographic assessment.

### 2.2. Measures

The Centre for Epidemiological Studies Depression Inventory—Revised (CESD-R) was utilized to evaluate the current level of depressive symptomatology, which closely reflects the criteria of Diagnostic and Statistical Manual of Mental Disorders, fourth edition (DSM-IV) for depression and demonstrates favorable psychometric properties [41]. It is an updated version of the CESD [42] and comprises 20 items with a five-point frequency score (0 = not at all or less than 1 day in the last week; 1:1–2 days in the last week; 2:3–4 days in the last week; 3:5–7 days in the last week; 4: nearly every day for two weeks). The higher the total score of the scale, the more severe the depressive symptomatology. The CESD-R is a screening tool for depression-related symptoms in the general population, making it particularly suitable for this study. All subjects in this study completed the scale rating before the experimental scan.

### 2.3. PET Data Acquisition and Preprocessing

The participants underwent cannulation of a minimum 22-gauge vein in each forearm, with the left arm being utilized for FDG infusion. ^18^FDG-PET data were acquired on a Siemens (Erlangen, Germany) 3 Tesla Biograph molecular MR (mMR) scanner (Syngo VB20 P) in list mode. The participants were positioned supine within the scanner bore, with their head placed in a 16-channel radiofrequency (RF) head coil. They were instructed to maintain as still a position as possible while keeping their eyes open and refraining from engaging in any particular thoughts, without any additional cognitive tasks or instructions provided. During the scan, ^18^ FDG was administered at an average dose of 233MBq via a BodyGuard 323 MR-compatible infusion pump (Caesarea Medical Electronics, Caesarea, Israel) at a rate of 36 mL/hr. The onset of infusion was synchronized with the start of the PET scan. ^18^FDG-PET (225) volumes were acquired after 95 min scanning; see Jamadar et al. [40] for detailed information.

The ^18^FDG-PET and T1-weighted (T1w) image were preprocessed using fMRIPrep. The T1w image was corrected for intensity non-uniformity (INU) with N4BiasFieldCorrection [43], distributed with ANTs 2.2.0 (RRID:SCR_004757) [44] and used as T1w-reference throughout the workflow. The T1w-reference was then skull-stripped with a Nipype implementation of the antsBrainExtraction.sh workflow (from ANTs), using OASIS30ANTs as target template. Volume-based spatial normalization to MNI152NLin2009cAsym standard space was performed through nonlinear registration with antsRegistration (ANTs 2.2.0), using brain-extracted versions of both T1w reference and the T1w template.

For each ^18^FDG-PET image, the following preprocessing was performed. First, a reference volume and its skull-stripped version were generated using a custom methodology of fMRIPrep. The PET reference was then co-registered to the T1w reference using bbregister (FreeSurfer 6.0.1), which implements boundary-based registration [45]. Co-registration was configured with nine degrees of freedom to account for distortions remaining in the PET reference. Head-motion parameters with respect to the PET reference (transformation matrices and six corresponding rotation and translation parameters) are estimated before any spatiotemporal filtering using mcflirt (FSL 5.0.9) [46]. The PET time-series was resampled into standard space, generating preprocessed PET data in MNI152NLin2009cAsym space, with a voxel size of 3 × 3 × 3 mm^3^.

### 2.4. Covariance Connectivity Analysis

The average temporal map of glucose metabolism was utilized to compute the PCC seed-based covariance connectivity, which was estimated using a previously proposed similarity algorithm for PET and ASL data [36,38,39]. The similarity (*S*) between PCC (region *i*) and the small spherical subsets (“searchlights”, region *j*) centered on each voxel in the brain is defined as follows:

(1) $$S=e^{\frac{-{\left(m_{i}-m_{j}\right)}^{2}}{2\left(\sigma_{i}^{2}+\sigma_{j}^{2}\right)}}$$

where m and σ denote the mean and standard deviation of the regional glucose metabolism, respectively.

**Figure 1:**
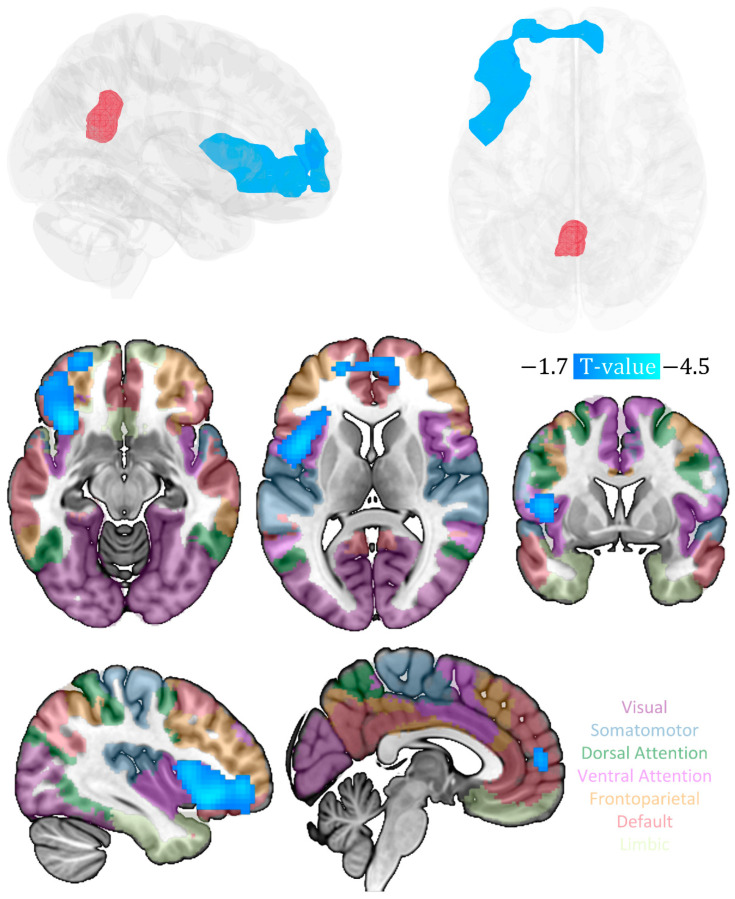
Spatial distribution of brain regions exhibiting significant negative correlation between PCC seed-based (red) metabolic connectivity and CESD-R. The hue of the translucent overlay on each slice corresponds to the Yeo 7-network [47] (indicated by the text color; visual network: dark purple; somatomotor network: gray blue; dorsal attention network: green; ventral attention network: light purple; frontoparietal network: yellow; default mode network: red).

To ensure the accuracy and precision of our region of interest (ROI), we constructed a PCC using data from a Neurosynth meta-analysis focused on depression (i.e., the term “depression”). To limit our analysis to relevant information, we applied a default threshold at FDR-corrected *p* < 0.01 based on Neurosynth’s recommendations (see Figure 1).

### 2.5. Statistical Analysis

The behavioral analyses were conducted using the R programming language (ver 4.2.3, https://www.r-project.org/, accessed on 6 August 2023). A significance level of *p* < 0.05, was applied to all statistical tests.

To delineate the metabolic covariance connectivity of PCC associated with a current level of depressive symptomatology, multiple regression analysis in SPM12 was conducted to investigate the metabolic covariance connectivity significantly linked with depression scales, with age, gender and mean framewise displacement as covariates. To correct for multiple comparisons, the cluster-level Family-Wise Error (FWE) correction was applied.

## 3. Results

The Chi-square test indicates a significant difference in the number of males and females included in the study (*p* = 0.012). After examining other variables, such as age, education level and CESD-R scores using independent *t*-tests, no significant disparities were found based on sex (*p*’s > 0.05, see Table 1). To control for the effect of sex on the relationship between PCC metabolic covariance connectivity and current level of depressive symptomatology, sex was included as a covariate in the multiple regression analysis.

We found a significant negative correlation between the CESD-R scale scores and the PCC metabolic covariance connectivity with multiple brain regions, particularly in the prefrontal areas, such as the anterior cingulate and medial prefrontal cortex, inferior and middle frontal gyrus (IFG and MFG), as well as insula (*p* < 0.05, cluster-level FWE correction; see Table 2 and Figure 1).

## 4. Discussion

In this study, we identified the metabolic covariance-based network of the PCC significantly correlated with depression symptomatology level in non-depressed young adults. The CESD-R scores were negatively correlated with the PCC metabolic connectivity in multiple brain regions, particularly in the prefrontal areas, such as the anterior cingulate, the medial prefrontal cortex, the inferior and middle frontal gyri, as well as insula. Notably, these regions are predominantly components of the default mode, salience and central executive networks, which are the main aberrant brain networks implicated in MDD pathology [48]. Our findings are consistent with the hypothesis suggesting that the metabolic covariance network of PCC, involving regions of CEN, SN and DMN, may potentially serve as neural biomarkers of increased risk for depression, which may be beneficial for early identification and intervention of individuals at risk for depression during young adulthood.

Firstly, our findings suggest that the PCC metabolic covariance connectivity can characterize the neurophysiological correlates of depression risk in young adults, facilitating early identification and prevention of this disease among youth. Over the past few decades, ^18^FDG-PET studies have consistently demonstrated the significance of cerebral glucose metabolism as a valid measure of neurophysiological activity in the brain for elucidating the neurophysiological basis of major depression. For instance, prior researches have consistently indicated that the patients with depression exhibit abnormal glucose metabolism in the regions including the prefrontal, anterior cingulate and posterior cingulate cortex, as well as the insula compared to healthy individuals [49,50,51,52]. A significant correlation between glucose metabolism in these brain areas and the severity of depression has been observed. To be specific, the subgenual prefrontal cortex shows a positive correlation between metabolic activity and depression severity. In contrast, in other regions, such as the posterior and lateral orbital cortex, anterior insula and ventrolateral prefrontal cortex, metabolic activity is negatively correlated with depressive ideation and severity ratings [52]. This suggests a compensatory response that modulates depressive symptoms. Although previous ^18^FDG-PET studies in the pathogenesis of MDD have demonstrated the crucial role of glucose metabolism in various brain regions, most investigations have primarily focused on the regional metabolic activation rather than interregional connectivity. More importantly, most participants in these studies were predominantly patients diagnosed with MDD, thus neglecting the exploration into healthy individuals without a depression diagnosis but showing variability in depressive symptomatology. In our study, focusing on the cohort of young and healthy adults, the metabolic covariance connectivity is based on the PCC, which mainly included extended areas of the prefrontal cortex and insula, and negatively correlated with the symptomatology level of depression in young adults.

The metabolic connectivity between the PCC and medial prefrontal cortex, major DMN nodes, is associated with cognitive function of young healthy adults [53]. Studies utilizing fMRI also offer additional evidence of abnormal connectivity between the PCC and the frontal regions of CEN, as well as insula, the core region of SN among young adults with MDD. These abnormalities have been documented to be associated with depressive rumination and impaired cognitive control [20,21]. Furthermore, the observed metabolic changes have been shown to be closely linked with depressive thoughts, depression severity, as well as treatment outcomes [6,7,9,10,49,50,54,55]. Moreover, abnormal metabolic activity in the anterior cingulate cortex was detected in young patients with MDD [56].A recent study using ^18^FDG-PET in combination with fMRI further suggested that the reduced metabolism of inferior frontal gyrus in MDD interacts with its impaired connectivity to other core regions [57]. Additionally, the insula, part of our PCC metabolic covariance connectivity results, has been found to exhibit abnormal inter-network coupling with both the DMN and CEN in MDD [58,59]. Considering that the prefrontal cortex and insula serve as central hubs of large-scale brain networks with diverse connectivity and dynamic interactions with various functional networks including the DMN [60,61,62], the coupling between the PCC and insula, prefrontal regions we observed is not unexpected.

Secondly, we applied the CESD-R to explore the association between PCC metabolic covariance network and the symptomatology level. Notably, unlike previous studies that typically utilized the BDI-I & II and HAMD scales designed for individuals with MDD, the CESD-R scale is specifically developed for assessing the current level of depressive symptomatology in the general population, with a particular focus on the affective component and depressed mood, core symptoms of MDD [63]. Of note, many other studies have investigated neuroimaging biomarkers of depression symptomatology based on the CESD-R [64,65,66,67]. Specifically, the functional connectivity strength between the PCC, the prefrontal cortex regions (dorsomedial and ventromedial prefrontal cortex) and the sgACC has been shown to negatively correlate with CESD-R scores in young healthy individuals [68]. At the network-level, the intra-brain interactions among large-scale brain networks, including the DMN, are also associated with depressive symptomatology as assessed by CESD-R in young adults without a diagnosed disease [69]. More importantly, abnormalities in the dynamic function of the default-frontoinsular network involving its posterior regions, the dorsolateral and medial prefrontal cortex, as well as insula are significantly associated with higher CESD-R scores and depressive rumination in healthy adolescents [70]. These findings may have some potential associations with our observation of the PCC metabolic covariance network involving the prefrontal regions and insula. Human brain metabolism exhibits an organized pattern of covariance, which is comparable to the corresponding fMRI resting-state time-series correlations [33]. The PCC metabolic covariance network we observed partially reflects functional connectivity as revealed by resting state BOLD.

In addition, the significant correlations of metabolic activation in prefrontal regions (dorsolateral and ventrolateral prefrontal cortex, superior frontal gyrus and orbital cortex), insula, cingulate gyrus and PCC with depressive symptoms has also been observed in previous research using HAMD and Montgomery-Asberg Rating Scale (MADRS) [9,13,49,50,51,54,71,72]. Moreover, MDD patients show a strong correlation between changes in PCC-insula/mid frontal cortex connectivity strength and HAMD scores [17,18], similarly to our results in nondepressed individuals in young adulthood. It is worth noting that these brain regions serve as crucial hubs within the default mode, salience and central executive networks—the triple networks implicated in MDD [48].

Within MDD, the DMN has been the most extensively investigated brain network, and the PCC, serving as the pivotal node of the DMN, has been found to be the sole node within the DMN that directly interacts with nearly all other nodes [73]. This observation is indicative for its crucial role in mediating intrinsic neuronal activities across the entire DMN. Aberrant connectivity between the PCC/precuneus (the core hubs of the posterior DMN) and the anterior cingulate and/or medial prefrontal area (the core hubs within the anterior subnetwork of DMN) has been associated with HAMA scores, rumination and response to antidepressant treatment [74,75,76,77]. Similar findings were also observed in subclinical depression in a young population, where altered connectivity between the PCC and sgACC, and between the precuneus and the dorsal medial prefrontal cortex was related to anhedonia and/or depression severity [23,78]. The metabolic connectivity between the PCC and medial prefrontal cortex in young healthy adults has also been demonstrated to be linked with cognitive task performance [53]. Another neuroimaging study has also shown that the functional connectivity of these three networks can predict current and future risk for depression in two independent young adult samples [19]. Reduced interregional connectivity between the DMN and CEN/SN [79,80,81,82] possibly contribute to the difficulty in moving from a self-referential state to a task-performing state in patients diagnosed with MDD. Given that the PCC is considered as a hub for integrating information from these three networks [83], our metabolic covariance network of PCC findings suggests that the PCC is involved in maintaining the balance among these multiple brain networks. Abnormalities between the PCC and other networks may be related to observed depressive symptomatology, and these abnormal correlations could be potential neural biomarkers that could be used for screening the risk of depression in the general population in young adulthood.

Finally, our study has several limitations, notably the relatively small sample size. Therefore, the present study should be viewed as merely an initial exploration based on metabolism for identifying neural correlates of depressive symptomatology levels in the healthy young population, and thus requires replication and validation in independent larger samples. Comparisons with data from other biological systems should be conducted, which holds promise toward the advancement of the disease risk screening in the young adults in the future. In addition, the present study is limited to the PCC as single seed region, and further investigation of other brain regions with metabolic abnormalities are warranted. Finally, disturbed sleep patterns as well as adverse childhood experiences are closely associated with the risk of developing depression, but this information was not collected. In follow-up studies, it would be useful to further investigate the influence of these possible confounding variables on our metabolic connectivity findings.

## 5. Conclusions

In conclusion, we delineated the metabolic covariance network of the PCC, involving regions of central executive network, salience network and default mode network, which correlated negatively with the level of depression symptomatology assessed with the CESD-R in healthy subjects in young adulthood. Our study emphasizes the importance of network-level brain features involved in the pathogenesis of MDD during young adulthood. Our results may further facilitate an early identification and intervention of at-risk individuals in the general population although further research is warranted to substantiate and reproduce our findings in large-scale clinical and non-clinical samples at risk. Because of the similarities with clinical depressed patients, our brain imaging observations also support the consideration of early psychotherapeutic interventions in non-clinical depressed samples potentially at risk to experience a depressive episode.

## Figures and Tables

**Table 1:** Demographic characteristics.

	Female	Male	*p*-Value
Count	20	7	0.012 ^1^
Age (years)	19.2 ± 1.196	20.14 ± 1.676	0.118 ^2^
Education (years)	14.55 ± 1.468	15.29 ± 1.496	0.267 ^2^
CESD-R	10.15 ± 8.952	6.14 ± 6.04	0.285 ^2^

^1^ Chi-square test. ^2^ Independent sample T test.

**Table 2:** The metabolic covariance connectivity of the PCC exhibited a significant correlation with CESD-R. (*P*_FWE-corr_: FWE corrected *p*-value).

Cluster	Anatomical Region	Cluster Size (Voxel)	Peak MNI Coordinates (x, y, z)	Peak t-Value (df = 22)	Cluster Level *P*_FWE-corr_
1		974			0.008
	inferior frontal gyrus	161	−33, 24, −6	−4.47	
	Insula	158	−33, 21, −9	−4.33	
	middle frontal gyrus	84	−39, 45, −3	−3.8	
	medial prefrontal cortex	72	12, 57, 3	−3.43	
	anterior cingulate	11	6, 54, 9	−3.18	

## Data Availability

The data presented in the study are available at https://doi.org/10.18112/openneuro.ds002898.v1.1.1, accessed on 6 August 2023.

## References

[1] Ferrari A.J., Charlson F.J., Norman R.E., Patten S.B., Freedman G., Murray C.J., Vos T., Whiteford H.A. (2013). Burden of depressive disorders by country, sex, age, and year: Findings from the global burden of disease study 2010. PLoS Med..

[2] Brody D.J., Pratt L.A., Hughes J.P. (2018). Prevalence of depression among adults aged 20 and over: United States, 2013–2016. NCHS Data Brief.

[3] Zisook S., Lesser I., Stewart J.W., Wisniewski S.R., Balasubramani G., Fava M., Gilmer W.S., Dresselhaus T.R., Thase M.E., Nierenberg A.A. (2007). Effect of age at onset on the course of major depressive disorder. Am. J. Psychiatry.

[4] Center for Behavioral Health Statistics and Quality (2015). Behavioral health trends in the United States: Results from the 2014 National Survey on Drug Use and Health (HHS Publication No. SMA 15-4927, NSDUH Series H-50). http://www.samhsa.gov/data/.

[5] Association A.C.H. (2019). American College Health Association-National College Health Assessment II: Reference Group Executive Summary Spring 2019.

[6] Jaracz J., Rybakowski J. (2002). Studies of cerebral blood flow in metabolism in depression using positron emission tomography (PET). Psychiatr. Pol..

[7] Drevets W.C. (2001). Neuroimaging and neuropathological studies of depression: Implications for the cognitive-emotional features of mood disorders. Curr. Opin. Neurobiol..

[8] Gusnard D.A., Raichle M.E. (2001). Searching for a baseline: Functional imaging and the resting human brain. Nat. Rev. Neurosci..

[9] Wei K., Xue H.-l., Guan Y.-h., Zuo C.-t., Ge J.-j., Zhang H.-y., Liu B.-j., Cao Y.-x., Dong J.-c., Du Y.-j. (2016). Analysis of glucose metabolism of 18F-FDG in major depression patients using PET imaging: Correlation of salivary cortisol and α-amylase. Neurosci. Lett..

[10] Drevets W.C., Bogers W., Raichle M.E. (2002). Functional anatomical correlates of antidepressant drug treatment assessed using PET measures of regional glucose metabolism. Eur. Neuropsychopharmacol..

[11] Buckner R.L., Andrews-Hanna J.R., Schacter D.L. (2008). The brain’s default network: Anatomy, function, and relevance to disease. Ann. N. Y. Acad. Sci..

[12] Raichle M.E., MacLeod A.M., Snyder A.Z., Powers W.J., Gusnard D.A., Shulman G.L. (2001). A default mode of brain function. Proc. Natl. Acad. Sci. USA.

[13] Brody A.L., Saxena S., Mandelkern M.A., Fairbanks L.A., Ho M.L., Baxter L.R. (2001). Brain metabolic changes associated with symptom factor improvement in major depressive disorder. Biol. Psychiatry.

[14] Mayberg H.S., Brannan S.K., Tekell J.L., Silva J.A., Mahurin R.K., McGinnis S., Jerabek P.A. (2000). Regional metabolic effects of fluoxetine in major depression: Serial changes and relationship to clinical response. Biol. Psychiatry.

[15] Goldapple K., Segal Z., Garson C., Lau M., Bieling P., Kennedy S., Mayberg H. (2004). Modulation of cortical-limbic pathways in major depression: Treatment-specific effects of cognitive behavior therapy. Arch. Gen. Psychiatry.

[16] Maddock R.J., Garrett A.S., Buonocore M.H. (2003). Posterior cingulate cortex activation by emotional words: fMRI evidence from a valence decision task. Hum. Brain Mapp..

[17] Zhu Z., Wang Y., Lau W.K., Wei X., Liu Y., Huang R., Zhang R. (2022). Hyperconnectivity between the posterior cingulate and middle frontal and temporal gyrus in depression: Based on functional connectivity meta-analyses. Brain Imaging Behav..

[18] Yang R., Gao C., Wu X., Yang J., Li S., Cheng H. (2016). Decreased functional connectivity to posterior cingulate cortex in major depressive disorder. Psychiatry Res. Neuroimaging.

[19] Bertocci M.A., Afriyie-Agyemang Y., Rozovsky R., Iyengar S., Stiffler R., Aslam H.A., Bebko G., Phillips M.L. (2023). Altered patterns of central executive, default mode and salience network activity and connectivity are associated with current and future depression risk in two independent young adult samples. Mol. Psychiatry.

[20] Zhu X., Wang X., Xiao J., Liao J., Zhong M., Wang W., Yao S. (2012). Evidence of a dissociation pattern in resting-state default mode network connectivity in first-episode, treatment-naive major depression patients. Biol. Psychiatry.

[21] Jacobs R.H., Jenkins L.M., Gabriel L.B., Barba A., Ryan K.A., Weisenbach S.L., Verges A., Baker A.M., Peters A.T., Crane N.A. (2014). Increased coupling of intrinsic networks in remitted depressed youth predicts rumination and cognitive control. PLoS ONE.

[22] Ely B.A., Xu J., Goodman W.K., Lapidus K.A., Gabbay V., Stern E.R. (2016). Resting-state functional connectivity of the human habenula in healthy individuals: Associations with subclinical depression. Hum. Brain Mapp..

[23] Philippi C.L., Motzkin J.C., Pujara M.S., Koenigs M. (2015). Subclinical depression severity is associated with distinct patterns of functional connectivity for subregions of anterior cingulate cortex. J. Psychiatr. Res..

[24] Benschop L., Poppa T., Medani T., Shahabi H., Baeken C., Leahy R.M., Pizzagalli D.A., Vanderhasselt M.-A. (2021). Electrophysiological scarring in remitted depressed patients: Elevated EEG functional connectivity between the posterior cingulate cortex and the subgenual prefrontal cortex as a neural marker for rumination. J. Affect. Disord..

[25] Chou T., Deckersbach T., Dougherty D.D., Hooley J.M. (2023). The default mode network and rumination in individuals at risk for depression. Soc. Cogn. Affect. Neurosci..

[26] Phillips A.A., Chan F.H., Zheng M.M.Z., Krassioukov A.V., Ainslie P.N. (2016). Neurovascular coupling in humans: Physiology, methodological advances and clinical implications. J. Cereb. Blood Flow Metab..

[27] Liu T.T. (2017). Reprint of ‘Noise contributions to the fMRI signal: An overview’. Neuroimage.

[28] Ward P.G., Orchard E.R., Oldham S., Arnatkevičiūtė A., Sforazzini F., Fornito A., Storey E., Egan G.F., Jamadar S.D. (2020). Individual differences in haemoglobin concentration influence BOLD fMRI functional connectivity and its correlation with cognition. NeuroImage.

[29] Logothetis N.K. (2008). What we can do and what we cannot do with fMRI. Nature.

[30] Magistretti P.J., Allaman I. (2015). A cellular perspective on brain energy metabolism and functional imaging. Neuron.

[31] Horwitz B., Duara R., Rapoport S.I. (1984). Intercorrelations of glucose metabolic rates between brain regions: Application to healthy males in a state of reduced sensory input. J. Cereb. Blood Flow Metab..

[32] Moeller J., Strother S., Sidtis J., Rottenberg D. (1987). Scaled subprofile model: A statistical approach to the analysis of functional patterns in positron emission tomographic data. J. Cereb. Blood Flow Metab..

[33] Di X., Biswal B.B., Alzheimer’s Disease Neuroimaging Initiative (2012). Metabolic brain covariant networks as revealed by FDG-PET with reference to resting-state fMRI networks. Brain Connect..

[34] Eidelberg D. (2009). Metabolic brain networks in neurodegenerative disorders: A functional imaging approach. Trends Neurosci..

[35] Niethammer M., Eidelberg D. (2012). Metabolic brain networks in translational neurology: Concepts and applications. Ann. Neurol..

[36] Wu G.-R., Baeken C. (2022). Individual interregional perfusion between the left dorsolateral prefrontal cortex stimulation targets and the subgenual anterior cortex predicts response and remission to aiTBS treatment in medication-resistant depression: The influence of behavioral inhibition. Brain Stimul..

[37] Wu G.-R., Baeken C. (2023). The left ventrolateral prefrontal cortex as a more optimal target for accelerated rTMS treatment protocols for depression?. Brain Stimul..

[38] Wu G.-R., Baeken C. (2023). Precision targeting in prediction for rTMS clinical outcome in depression: What about sgACC lateralization, metabolic connectivity, and the potential role of the cerebellum?. Eur. Arch. Psychiatry Clin. Neurosci..

[39] Wu G.-R., Baeken C. (2023). Lateralized subgenual ACC metabolic connectivity patterns in refractory melancholic depression: Does it matter?. Cereb. Cortex.

[40] Jamadar S.D., Ward P.G., Close T.G., Fornito A., Premaratne M., O’Brien K., Stäb D., Chen Z., Shah N.J., Egan G.F. (2020). Simultaneous BOLD-fMRI and constant infusion FDG-PET data of the resting human brain. Sci. Data.

[41] Eaton W.W., Muntaner C., Smith C., Tien A., Ybarra M. (2004). Center for epidemiologic studies depression scale: Review and revision. The Use of Psychological Testing for Treatment Planning and Outcomes Assessment.

[42] Radloff L.S. (1977). The CES-D scale: A self-report depression scale for research in the general population. Appl. Psychol. Meas..

[43] Tustison N.J., Avants B.B., Cook P.A., Zheng Y., Egan A., Yushkevich P.A., Gee J.C. (2010). N4ITK: Improved N3 bias correction. IEEE Trans. Med. Imaging.

[44] Avants B.B., Epstein C.L., Grossman M., Gee J.C. (2008). Symmetric diffeomorphic image registration with cross-correlation: Evaluating automated labeling of elderly and neurodegenerative brain. Med. Image Anal..

[45] Greve D.N., Fischl B. (2009). Accurate and robust brain image alignment using boundary-based registration. Neuroimage.

[46] Jenkinson M., Bannister P., Brady M., Smith S. (2002). Improved optimization for the robust and accurate linear registration and motion correction of brain images. Neuroimage.

[47] Thomas Yeo B., Krienen F.M., Sepulcre J., Sabuncu M.R., Lashkari D., Hollinshead M., Roffman J.L., Smoller J.W., Zöllei L., Polimeni J.R. (2011). The organization of the human cerebral cortex estimated by intrinsic functional connectivity. J. Neurophysiol..

[48] Menon V. (2011). Large-scale brain networks and psychopathology: A unifying triple network model. Trends Cogn. Sci..

[49] Mah L., Zarate C.A., Singh J., Duan Y.-F., Luckenbaugh D.A., Manji H.K., Drevets W.C. (2007). Regional cerebral glucose metabolic abnormalities in bipolar II depression. Biol. Psychiatry.

[50] Drevets W.C. (1999). Prefrontal cortical-amygdalar metabolism in major depression. Ann. N. Y. Acad. Sci..

[51] Videbech P. (2000). PET measurements of brain glucose metabolism and blood flow in major depressive disorder: A critical review. Acta Psychiatr. Scand..

[52] Drevets W.C. (2000). Neuroimaging studies of mood disorders. Biol. Psychiatry.

[53] Yakushev I., Chételat G., Fischer F.U., Landeau B., Bastin C., Scheurich A., Perrotin A., Bahri M.A., Drzezga A., Eustache F. (2013). Metabolic and structural connectivity within the default mode network relates to working memory performance in young healthy adults. Neuroimage.

[54] Drevets W.C., Videen T.O., Price J.L., Preskorn S.H., Carmichael S.T., Raichle M.E. (1992). A functional anatomical study of unipolar depression. J. Neurosci..

[55] Dunlop B.W., Kelley M.E., McGrath C.L., Craighead W.E., Mayberg H.S. (2015). Preliminary findings supporting insula metabolic activity as a predictor of outcome to psychotherapy and medication treatments for depression. J. Neuropsychiatry Clin. Neurosci..

[56] He J., Wang D., Ban M., Kong L., Xiao Q., Yuan F., Zhu X. (2022). Regional metabolic heterogeneity in anterior cingulate cortex in major depressive disorder: A multi-voxel 1H magnetic resonance spectroscopy study. J. Affect. Disord..

[57] Su H., Zuo C., Zhang H., Jiao F., Zhang B., Tang W., Geng D., Guan Y., Shi S. (2018). Regional cerebral metabolism alterations affect resting-state functional connectivity in major depressive disorder. Quant. Imaging Med. Surg..

[58] Ge R., Hassel S., Arnott S.R., Davis A.D., Harris J.K., Zamyadi M., Milev R., Frey B.N., Strother S.C., Müller D.J. (2021). Structural covariance pattern abnormalities of insula in major depressive disorder: A CAN-BIND study report. Prog. Neuro-Psychopharmacol. Biol. Psychiatry.

[59] Lu F., Cui Q., Chen Y., He Z., Sheng W., Tang Q., Yang Y., Luo W., Yu Y., Chen J. (2023). Insular-associated causal network of structural covariance evaluating progressive gray matter changes in major depressive disorder. Cereb. Cortex.

[60] Kurth F., Zilles K., Fox P.T., Laird A.R., Eickhoff S.B. (2010). A link between the systems: Functional differentiation and integration within the human insula revealed by meta-analysis. Brain Struct. Funct..

[61] Bressler S.L., Menon V. (2010). Large-scale brain networks in cognition: Emerging methods and principles. Trends Cogn. Sci..

[62] Ainsworth M., Wu Z., Browncross H., Mitchell A.S., Bell A.H., Buckley M.J. (2022). Frontopolar cortex shapes brain network structure across prefrontal and posterior cingulate cortex. Prog. Neurobiol..

[63] Heo E.-H., Choi K.-S., Yu J.-C., Nam J.-A. (2018). Validation of the center for epidemiological studies depression scale among Korean adolescents. Psychiatry Investig..

[64] Hwang J., Egorova N., Yang X., Zhang W., Chen J., Yang X., Hu L., Sun S., Tu Y., Kong J. (2015). Subthreshold depression is associated with impaired resting-state functional connectivity of the cognitive control network. Transl. Psychiatry.

[65] Beevers C.G., Clasen P., Stice E., Schnyer D. (2010). Depression symptoms and cognitive control of emotion cues: A functional magnetic resonance imaging study. Neuroscience.

[66] Zhang X., Di X., Lei H., Yang J., Xiao J., Wang X., Yao S., Rao H. (2016). Imbalanced spontaneous brain activity in orbitofrontal-insular circuits in individuals with cognitive vulnerability to depression. J. Affect. Disord..

[67] Sun H., Luo L., Yuan X., Zhang L., He Y., Yao S., Wang J., Xiao J. (2018). Regional homogeneity and functional connectivity patterns in major depressive disorder, cognitive vulnerability to depression and healthy subjects. J. Affect. Disord..

[68] Strikwerda-Brown C., Davey C.G., Whittle S., Allen N.B., Byrne M.L., Schwartz O.S., Simmons J.G., Dwyer D., Harrison B.J. (2015). Mapping the relationship between subgenual cingulate cortex functional connectivity and depressive symptoms across adolescence. Soc. Cogn. Affect. Neurosci..

[69] Schultz D.H., Ito T., Solomyak L.I., Chen R.H., Mill R.D., Anticevic A., Cole M.W. (2018). Global connectivity of the fronto-parietal cognitive control network is related to depression symptoms in the general population. Netw. Neurosci..

[70] Kaiser R.H., Kang M.S., Lew Y., Van Der Feen J., Aguirre B., Clegg R., Goer F., Esposito E., Auerbach R.P., Hutchison R.M. (2019). Abnormal frontoinsular-default network dynamics in adolescent depression and rumination: A preliminary resting-state co-activation pattern analysis. Neuropsychopharmacology.

[71] Baxter L.R., Schwartz J.M., Phelps M.E., Mazziotta J.C., Guze B.H., Selin C.E., Gerner R.H., Sumida R.M. (1989). Reduction of prefrontal cortex glucose metabolism common to three types of depression. Arch. Gen. Psychiatry.

[72] Martinot J.-L., Hardy P., Feline A., Huret J.-D., Mazoyer B., Attar-Levy D., Pappata S., Syrota A. (1990). Left prefrontal glucose hypometabolism in the depressed state: A confirmation. Am. J. Psychiatry.

[73] Fransson P., Marrelec G. (2008). The precuneus/posterior cingulate cortex plays a pivotal role in the default mode network: Evidence from a partial correlation network analysis. Neuroimage.

[74] Goldstein-Piekarski A.N., Staveland B.R., Ball T.M., Yesavage J., Korgaonkar M.S., Williams L.M. (2018). Intrinsic functional connectivity predicts remission on antidepressants: A randomized controlled trial to identify clinically applicable imaging biomarkers. Transl. Psychiatry.

[75] Chen Y., Wang C., Zhu X., Tan Y., Zhong Y. (2015). Aberrant connectivity within the default mode network in first-episode, treatment-naive major depressive disorder. J. Affect. Disord..

[76] Peng X., Wu X., Gong R., Yang R., Wang X., Zhu W., Lin P. (2021). Sub-regional anterior cingulate cortex functional connectivity revealed default network subsystem dysfunction in patients with major depressive disorder. Psychol. Med..

[77] Berman M.G., Peltier S., Nee D.E., Kross E., Deldin P.J., Jonides J. (2011). Depression, rumination and the default network. Soc. Cogn. Affect. Neurosci..

[78] Rzepa E., McCabe C. (2018). Anhedonia and depression severity dissociated by dmPFC resting-state functional connectivity in adolescents. J. Psychopharmacol..

[79] Manoliu A., Meng C., Brandl F., Doll A., Tahmasian M., Scherr M., Schwerthöffer D., Zimmer C., Förstl H., Bäuml J. (2014). Insular dysfunction within the salience network is associated with severity of symptoms and aberrant inter-network connectivity in major depressive disorder. Front. Hum. Neurosci..

[80] Zheng H., Xu L., Xie F., Guo X., Zhang J., Yao L., Wu X. (2015). The altered triple networks interaction in depression under resting state based on graph theory. BioMed Res. Int..

[81] Guha A., Yee C.M., Heller W., Miller G.A. (2021). Alterations in the default mode-salience network circuit provide a potential mechanism supporting negativity bias in depression. Psychophysiology.

[82] Chand G.B., Wu J., Hajjar I., Qiu D. (2017). Interactions of the salience network and its subsystems with the default-mode and the central-executive networks in normal aging and mild cognitive impairment. Brain Connect..

[83] Li R., Zhang S., Yin S., Ren W., He R., Li J. (2018). The fronto-insular cortex causally mediates the default-mode and central-executive networks to contribute to individual cognitive performance in healthy elderly. Hum. Brain Mapp..

